# Risk Factors Associated With Coronary Disease in Saudi Arabia: A Comprehensive Review

**DOI:** 10.7759/cureus.79123

**Published:** 2025-02-16

**Authors:** Abdulazeez A Alsuheimy, Ali B Aljuaid, Falha N Albalawi, Eman Hassounah, Fawaz Modahi, Khalid Alkhurayji

**Affiliations:** 1 Medicine, Royal College of Surgeons, Dublin, IRL; 2 Dental Center, Prince Sultan Military Medical City, Riyadh, SAU; 3 Dental Hygiene, King Abdulaziz Medical City, Riyadh, SAU; 4 Health Information Management and Technology, Imam Abdulrahman Bin Faisal University, Dammam, SAU

**Keywords:** acute cardiac care, cardiology, cardiovascular disease, health care outcomes, heart

## Abstract

Coronary artery disease (CAD) has become a major health challenge across the globe and is one of the major causes of mortality and morbidity in several countries. In Saudi Arabia, recent studies have emphasized the burden of CAD. Therefore, in this study, we aim to assess the risk factors associated with CAD in Saudi Arabia. The Population, Intervention, Comparison, and Outcome (PICO) model was used to refine the research question, which defined patients with CAD and identified risk factors and prevention strategies. PubMed, Web of Science, and Google Scholar were searched from January 1, 2010, until December 1, 2024. The common risk factors were diabetes, hypertension, obesity, and smoking, indicating their significant impact on health outcomes and the urgency for focus prevention strategies such as education and routine clinical screening. The risk factors contributing to CAD emphasize the need for targeted public health intervention and improvement in the management of risk factors in addition to specific health education content to improve healthy habits in the community.

## Introduction and background

Coronary artery disease (CAD) has emerged as a major global health concern, accounting for a sizable proportion of mortality and morbidity in many countries [1]. The World Health Organization (WHO) estimates that 17 million people die each year from CAD, which accounts for around 31% of total population deaths [2]. These concerning findings highlighted the necessity for researchers to act and develop focused prevention efforts to combat CAD [3].

Several conditions can impact the heart and blood vessels, including heart failure, arrhythmias, stroke, and CAD [4]. These disorders usually arise due to complex interactions between various risk factors, which can be classified into modifiable and non-modifiable components. These risk factors vary in terms of contribution to CAD. Modifiable risk factors include health habits, smoking, a poor diet, physical inactivity, and alcohol consumption and can increase the prevalence of CAD [5]. Other risk factors include medical issues such as hypertension, dyslipidemia, and diabetes [6]. Non-modifiable risk factors such as age, gender, and genetic predisposition are thought to have a significant effect on an individual's overall health. Previous studies have demonstrated that as age increases, the risk of CAD also increases [7].

In recent years, the prevalence of CAD risk factors has increased, especially in regions experiencing major urbanization and lifestyle changes. For instance, in Saudi Arabia, the prevalence rate of diabetes reached more than 24% among adults, while the prevalence rate of smoking tobacco reached more than 16% among adults. The transition from traditional to more sedentary lifestyles, along with excessive food consumption, has resulted in a large increase in obesity rates and associated health problems [8,9]. These changes prompted a need for immediate public health intervention to reverse these trends and enhance CAD health outcomes [10]. Recent studies have emphasized the burden of CAD in Saudi Arabia, with key contributors including rising obesity, smoking rates, and a lack of understanding [11]. Many patients are unaware of their risk factors or the significance of adopting a healthy lifestyle in preventing CAD [12]. Stress and worry can also have an impact on CAD. Several investigations have shown that mental health has a significant role in heart disease, with persistent stress leading to hypertension [13].

There is a shortage of knowledge in this field, necessitating reviews from current studies in the context of Saudi Arabia. Therefore, this review seeks to synthesize current research findings on the risk factors associated with cardiovascular disease. This research aims to provide a comprehensive review of cardiovascular health in Saudi Arabia, taking into account both modifiable and non-modifiable factors. Furthermore, it seeks to identify effective prevention strategies that can be used at both the individual and community levels to lower the risks associated with CAD. By raising knowledge of these critical variables, healthcare providers and policymakers can devise targeted programs that can encourage healthier lifestyles and, eventually, reduce the burden of cardiovascular disease in the population.

## Review

Methods

The Population, Intervention, Comparison, and Outcome (PICO) model was used to refine the following research question: what are the risk factors associated with CAD among patients? (Table 1).

**Table 1:** PICO model

Items	Terms
Population (P)	Patients with CAD
Intervention (I)	Identification and analysis of risk factors (e.g., lifestyle, genetic predisposition, comorbidities)
Comparison (C)	Not applicable
Outcome (O)	Identification of targeted prevention strategies

Eligibility criteria

The purpose of this review was to identify, evaluate, and synthesize observational studies (of all types), case-control studies, cohort studies, and cross-sectional studies that examined risk factor identification and analysis (e.g., lifestyle, genetic predisposition, comorbidities). The review included studies on adults in Saudi Arabia. The primary outcomes of the review were the identification of common risk factors, creation of targeted preventative methods, and reduction in the incidence of CAD.

Search strategy

We searched the following databases: PubMed, Web of Science, and Google Scholar. Databases were searched from January 1, 2010, to December 1, 2024 (Appendix). We limited our search to exclude specific types of publications. Conference abstracts, articles in press, theses, and books or book chapters were specifically excluded from the search results. We limited our search to the English language. We personally examined the reference lists of the listed studies and contacted experts.

Screening

The authors independently examined titles and abstracts against inclusion criteria. The full texts of publications determined to be eligible following screening were obtained. The citation search was checked. Consensus meetings were used to reach agreement for the inclusion and exclusion of studies.

Data extraction

A data extraction form was used to extract the study characteristics and outcome data, which was piloted on five studies to enhance data extraction and usability of the form. No modification was done based on the pilot extraction. Multiple authors extracted the data, and censuses was used to reach agreement for the extraction to ensure consistency. The author of this study gathered the following information from the included study types: observational studies (all types), case-control studies, cohort studies, and cross-sectional studies. The data extracted from each study include study authors, year in which the study was published, study design, geographical location, number of participants, risk factor of CAD, summary outcome of the study, and preventative solutions.

Results

**Figure 1:**
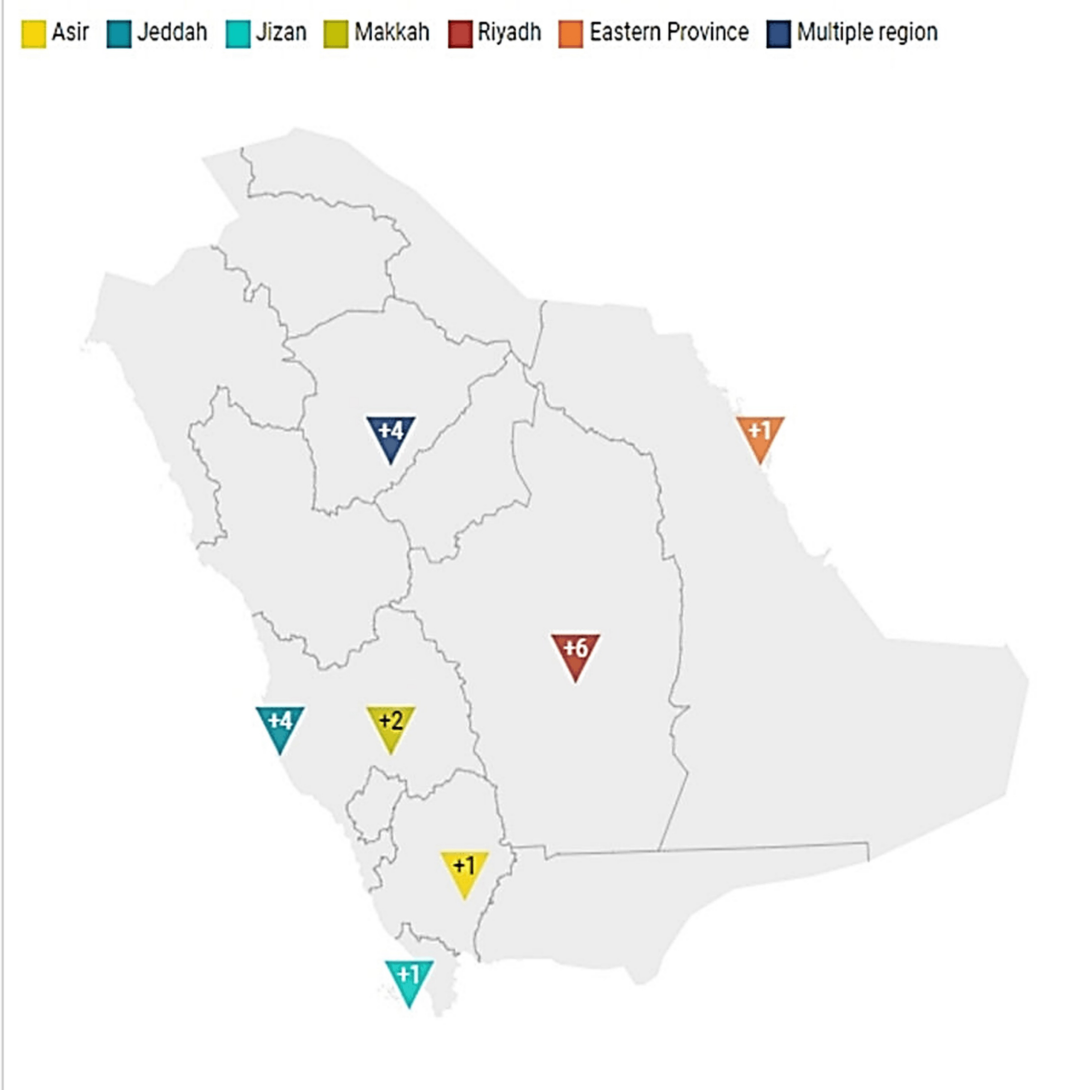
Studies distribution across Saudi Arabia's regions Author’s creation using https://www.datawrapper.de/

**Figure 2:**
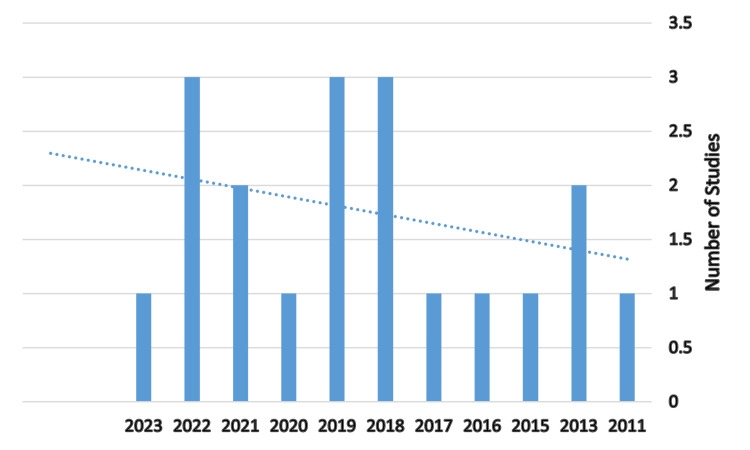
Research activity across years

Figure 1 shows the distribution of research across geographical places, with Riyadh having the most studies, followed by Jeddah, representing patterns and biases across studies across Saudi Arabia regions. Figure 2 illustrates that the studies and research activities ranged from one to three studies every year, with the majority of them being one study per year.

Table 2 summarizes the primary findings from CAD. These findings show the interplay of risk factors, summary outcome, and prevention approaches, providing important insights into the region's public health issues.

**Table 2:** Characteristics of studies included, risk factors, summary outcome, and prevention strategies ACS, acute coronary syndrome; BMI, body mass index; CAD, coronary artery disease; CAC, coronary artery; CKD, chronic kidney disease; DM, diabetes mellitus; HTN, hypertension; IHD, ischemic heart disease; OR, odds ratio; PPI, proton pump inhibitor calcification

Reference	Region	Research design	Risk factor	Summary outcome	Prevention strategies
[14]	Jeddah	Retrospective	Smoking, overweight, obesity, and dyslipidemia	The majority of patients (67%) underwent percutaneous coronary intervention, and there were no adverse events reported during hospitalization or within 30 days post-discharge.	The study emphasizes the need for preventive measures targeting modifiable risk factors, particularly smoking cessation and lifestyle changes
[15]	Multiple regions	Retrospective	Age and sex (older males were more prevalent with CAD); history of IHD, DM, and dyslipidemia; lower prevalence of chronic kidney disease in patients with CAD	The study found that CAD patients had significantly higher unadjusted hospital and 30-day mortality rates compared to non-CAD patients.	Better management of risk factors and more aggressive treatment protocols for those presenting with ACS
[16]	Jeddah	Case-control	Current smoking (OR: 4.5), excessive body weight (OR: 2.99), dyslipidemia (OR: 2.51). HTN, DM, and moderate-to-high physical activity were also associated with CAD	The study concluded that there was no significant association between the use of PPIs and the occurrence of a first non-fatal ACS event (p>0.05).	Need for continued health education and promotion to modify known risk factors influencing the occurrence of ACS
[17]	Jeddah	Retrospective	Higher prevalence of DM among Saudis, differences; non-Saudis were noted to have higher levels of certain biomarkers, which may be linked to delayed healthcare access	Non-Saudis had poorer outcomes related to healthcare access and insurance coverage, leading to higher rates of mortality and major adverse cardiac events	Improved healthcare access and insurance coverage for expatriates to ensure timely treatment of ACS
[18]	Riyadh	Retrospective	DM, HTN, hypercholesterolemia, and a family history of CAD	The main outcome of the study indicated that there was no significant association between osteoporosis and higher CAC scores.	Lifestyle modifications, regular screening, management of risk factor
[11]	Riyadh	Cross-sectional	Smoking, overweight/obesity (measured by BMI), physical inactivity, and consumption of fast foods	The findings revealed that more than one-fourth of participants could not identify any CAD risk factors and around one-third had more than two risk factors.	To address the low awareness and high prevalence of CAD risk factors, the study suggests implementing public health awareness programs and cardiac educational activities
[19]	Multiple regions	Cross-sectional	Dyslipidemia, abdominal obesity, HTN, DM, and smoking	High prevalence of cardiovascular risk factors among the Saudi population, with significant portions of patients having poor control of these risk factors. For instance, HTN prevalence increased from 26.1% in previous studies to over 40% in this study, indicating a growing public health concern.	Community-based screening for cardiovascular risk factors, increased awareness and education on healthy lifestyle choices, including diet and exercise
[20]	Makkah	Retrospective	DM, CKD, and other traditional risk factors such as HTN, hyperlipidemia, and smoking were also assessed	The presence of left ventricular thrombus was also more common in group I, indicating a higher risk of adverse outcomes.	Regular screening and management of DM and kidney function. Lifestyle modifications (diet, exercise) to reduce cardiovascular risk. Medical treatment strategies tailored to the individual risk profiles of patients with ACS.
[21]	Riyadh	Retrospective	DM, HTN, hypercholesterolemia, family history of CAD, and obesity (defined as a BMI >30 kg/m²)	The study found that age and DM were identified as significant independent predictors of the severity of CAC	It emphasized the importance of using CAC score for better risk stratification, prevention, and treatment of CAD, which is a leading cause of death among females
[22]	Multiple regions	Cross-sectional	Obesity, high blood pressure, anxiety, stress, smoking, and family history of cardiovascular diseases	The findings indicated a generally unsatisfactory level of knowledge regarding CAD risk factors among participants. For instance, 57.8% lacked knowledge about the complications of CAD and 55% were unaware of the primary drugs used to treat it.	To improve awareness and reduce the risk of CAD, the study emphasizes the importance of public health education
[23]	Jeddah	Retrospective	DM, dyslipidemia, and HTN	The study found that 99.5% of patients recovered and were discharged without adverse hospital events. However, the most common adverse event was reinfarction, occurring in 17.6% of patients, which was strongly associated with HTN and DM.	The study emphasizes the need for greater attention to preventive strategies, including lifestyle changes and evidence-based treatments for cardiovascular risk factors
[24]	Makkah	Case-control	High blood pressure (OR = 2.32), high blood sugar levels (OR = 1.90), high cholesterol levels (OR = 1.82), excessive BMI (OR = 1.73)	The study concluded that the probability of developing CAD when having the four significant risk factors simultaneously was approximately 85.69%. High blood pressure was identified as the most common and influential risk factor.	Regular health screenings to monitor blood pressure, blood sugar, and cholesterol levels. Promoting a healthy lifestyle, including a balanced diet and regular physical activity to manage weight and reduce BMI. Education on the importance of managing chronic conditions and lifestyle choices, particularly for high-risk populations like Hajj pilgrims.
[25]	Riyadh	Cross-sectional	High blood pressure, DM, physical inactivity, smoking, obesity overweight status, HTN, and lack of knowledge regarding CAD	The study concluded that Saudi adults possess adequate knowledge of CAD risk factors and prevention strategies	Educating the public about risk factors and management of CVD, promoting lifestyle changes such as increased physical activity and healthy eating, encouraging smoking cessation, and raising awareness through continuous health education initiatives
[26]	Aseer	Retrospective	DM, HTN, smoking, hyperlipidemia, and family history of IHD	No significant differences in in-hospital mortality or re-infarction rates between genders were observed. Female patients with ACS tended to be older than male patients.	Increased awareness and education about the risk factors for ACS, particularly in women, to address the lower rates of smoking and hyperlipidemia. Implementation of gender-sensitive approaches in the management and treatment of ACS to ensure equitable care.
[27]	Jizan	Retrospective	DM, HTN, smoking, family history, dyslipidemia, obesity, and low physical activity	Smoking was a major risk factor, particularly in males. There was no significant gender difference in CAD severity; however, the number of obstructed vessels was associated with poor prognosis in both genders.	Increased awareness and education regarding CAD vulnerability, especially among women. Equal enrollment of females and males in cardiovascular trials to ensure comprehensive understanding and treatment. Targeted preventative interventions to reduce the financial burden and negative outcomes associated with CAD.
[28]	Multiple regions	Retrospective	DM, HTN, hyperlipidemia, and a history of CAD	The study highlighted significant gender differences in the presentation and outcomes of CAD. Women were more likely to present with non-ST-segment elevation myocardial infarction and unstable angina	The study suggests the need for increased awareness among healthcare providers regarding the prompt administration of effective therapies,
[29]	Riyadh	Retrospective	Age, gender, DM, HTN, hypercholesterolemia, family history of CAD, obesity, and smoking	The study revealed a high prevalence of (55%) in patients with normal myocardial perfusion imaging, with 12% of patients exhibiting severe coronary atherosclerosis (CAC score > 300)	The study emphasizes the need for better detection of subclinical coronary atherosclerosis in patients.
[30]	Riyadh	Cross-sectional	Smoking, lack of exercise, obesity, HTN, high levels of cholesterol, and genetic predisposition to heart diseases	The study indicated a significant lack of knowledge and poor lifestyle practices among the participants. Notably, 46.1% of participants believed that CAD can affect individuals under the age of 45, while a substantial portion of the population exhibited unhealthy lifestyle choices.	The study emphasizes the need for health authorities to adopt a more targeted approach to increase awareness about CAD and its risk factors.
[31]	Eastern Province	Case-control	HTN and DM	First to report the association of these polymorphisms with CAD in the population of the Eastern Province of Saudi Arabia. The rs5882 polymorphism (CETP) showed a significant association and therefore could be a promising marker for CAD risk estimation, while the rs708272 polymorphism had a protective effect from CAD	Understanding genetic risk factors can inform targeted prevention and management strategies for CAD in at-risk populations

Table 3 gives the distribution of studies within the research design in this review. Researchers used the retrospective design (57.89%), followed by the cross-sectional design (26.32%) and case-control studies (15.79%), representing a high pattern across the retrospective design.

**Table 3:** research types across the review N, number; %, percentage

Research design	Reference	N (%)
Retrospective	[14], [15], [17], [18], [20], [21], [23], [26-29]	11 (57.89)
Case-control	[16], [24], [31]	3 (15.79)
Cross-sectional	[11], [19], [22], [25], [30]	5 (26.32)
Total		19 (100)

Prevalence of risk factors

The studies identify a number of modifiable risk factors for CAD, including high levels of smoking, obesity, diabetes, and hypertension in multiple locations. For instance, knowledge of smoking as a risk factor for CAD was high in several studies [25,26].

Gender and demographic differences

The disturbing association between risk variables and poor health outcomes was discovered, along with an increase in mortality rates and CAD complications. For instance, males were found to have CAD at a higher rate than females, implying that demographic vulnerability may demand individualized interventions. The results also showed that timely intervention can significantly improve outcomes through the management of risk factors and improvement of prevention strategies. The table shows significant gender disparities in the presentation and outcome of CAD. Women were found to present with distinct forms of myocardial infarctions than men, highlighting the need for gender-specific preventive treatments. Furthermore, the gap in results between Saudis and non-Saudis reveals inconsistencies in healthcare services that must be addressed in order to provide equal care.

Need for comprehensive prevention strategies

Several intervention and prevention attempts emphasized lifestyle modifications, public health education, and better chronic illness management. In fact, the studies called for community-based screening and health education initiatives, which are crucial given the prevalence of these risk factors and the clear lack of awareness. Notably, many survey participants reported a lack of understanding of CAD risk factors, highlighting the need for targeted educational programs to bridge the knowledge gap.

Healthcare access and policy implications

The important need for enhanced healthcare access and insurance coverage spurred politicians to look into ways to increase healthcare access, such as community outreach programs and health insurance reforms that prioritize cardiovascular health.

Public health concerns

The overall findings indicate that CAD is a growing public health concern in Saudi Arabia. The rising prevalence and accompanying consequences necessitated continuing research and surveillance in order to adequately address these risk factors. Stakeholders can help reduce the prevalence of CAD in the population by emphasizing prevention, awareness, and equitable healthcare access. Continuous research, policymaking, and community participation are essential to enhance the health outcomes and quality of life for persons at risk of cardiovascular disease.

**Figure 3:**
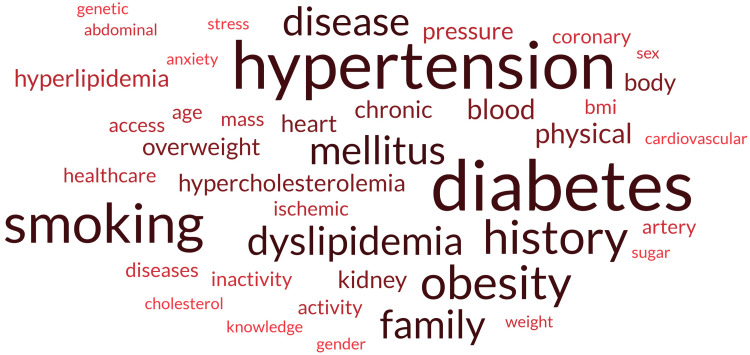
Word cloud represents the frequency of risk factors among the studies Created by author using https://www.freewordcloudgenerator.com/

A glossary of terms related to cardiovascular health and associated risk factors is shown in Figure 3. Each phrase highlights key themes that were most likely identified by a study of studies related to CAD. The word cloud was generated based on the frequency of each risk factor identified in this review. The terms were captured from the studies' data extraction forms that were part of this review. The greater the frequency of a term, the larger it becomes.

Commonly occurring risk factors

Diabetes, hypertension, obesity, and smoking were identified as major risk factors related to CAD. Their prominence in the text highlighted their major prevalence and impact on health outcome. Nonetheless, hyperlipidemia (abnormally high level of fats), dyslipidemia (lipid in body out of balance), and cholesterol illustrate that the differences in lipid metabolism and the recognition of the risk of CAD highlight the need of monitoring and controlling patients' cholesterol levels. Furthermore, lifestyle choices related to inactivity and physical activity, particularly sedentary behaviors, can be identified as risk factors. For instance, increased physical activity has been associated with improved health outcomes [27].

Cardiovascular, ischemic, and coronary diseases emphasize the need for focused interventions on heart-related diseases, underlining the review's importance in comprehending prevention types of heart ailments including ischemic heart disease. Moreover, the inclusion of chronic conditions emphasizes the long-term nature of numerous cardiovascular illnesses. The term "family history" implies a genetic predisposition to heart disease, whereas "access" refers to the importance of healthcare services access in managing cardiovascular illnesses. Certain demographics were observed that influence CAD health. Understanding how age and sex impact the risk variables can help in customized prevention measures and treatment options. For instance, as age increases, the risk of CAD also increases. Also, psychological components such as chronic stress were found to play a vital impact in mental health status as well as overall cardiovascular health [22]. The absence of awareness and knowledge of the risk factors, in particular stress and worry, can lead to poor outcomes.

General health

The multifaceted nature of CAD represents the significant representation of health condition, risk factors, demographic factors, and psychological aspects related to health outcome. The fundamental health indictors that are commonly measured in clinical settings and are crucial for assessing patient health and risk factors for CAD were BMI, blood pressure, and cholesterol levels. Demographics such as age and psychological factors such as chronic stress can lead to CAD, resulting in multifaceted associations. This interpretation lays the groundwork for additional research into how these factors interact and the implications for public health measures targeted at lowering the burden of heart disease in populations. Understanding these factors can aid healthcare providers in developing targeted preventative and intervention strategies to improve cardiovascular health outcomes.

Discussion

This research revealed various risk factors for CAD, emphasizing the importance of improving patients' health through direct interventions such as education, good habits, and regular screening. The most common risk factors observed in Saudi Arabia were smoking, diabetes, and hypertension, stressing the need for controlling these factors to avoid CAD. By merging data from many studies conducted in various areas across Saudi Arabia, a better knowledge of the complicated interplay of lifestyle, genetics, and socioeconomic factors that contribute to CAD has emerged.

The study's findings found an increase in risk factors such as smoking, obesity, and physical inactivity. This study validates prior research that has shown that poor lifestyle promotes the development of CAD [32,33]. To demonstrate this argument, a study conducted across multiple populations identified several trends indicating that smoking and obesity can be significant risk factors for CAD [34-36]. Furthermore, lifestyle factors may influence chronic disorders including hypertension and diabetes. In fact, several studies have shown that controlling these conditions can help prevent CAD [37]. Nonetheless, family history also plays a major role in the development of CAD [18].

Diabetes and hypertension have been identified as major risk factors in Saudi Arabia and other countries, emphasizing the need for integrated healthcare techniques aimed at early detection and management of these disorders. Given that certain barriers were observed between rural and urban regions in terms of healthcare services availability [38,39], the review emphasizes the importance of socioeconomic determinants such as healthcare access and education [40]. Many studies have found that lower socioeconomic level is frequently related to increased prevalence of CAD risk variables and poorer health outcomes [41]. This is especially pertinent in Saudi Arabia, where discrepancies in healthcare access can result in considerable differences in the management of cardiovascular risk factors [42, 43]. The findings from this review suggest that addressing these disparities through targeted healthcare policies and community education initiatives is vital for improving cardiovascular health outcomes.

Stress and anxiety can play a significant role in CAD. Previous research has shown that mental health is inextricably linked to physical health and that long-term stress can lead to hypertension and other cardiac disorders [44]. This study is consistent with previous research suggesting the importance of incorporating mental health in CAD prevention, which has revealed improvements in patients’ quality of life [45].

This review provides a comprehensive summary of the risk factors associated with CAD, emphasizing the significance of treating both modifiable and non-modifiable factors. This review is consistent with existing literature, indicating the need for multifaceted intervention and preventative strategies that include lifestyle modification, disease management, and social and mental health assistance [46,47].

Healthcare providers and policymakers must work more closely to solve the existing situation and the growing burden of CAD. This will collectively inform more effective interventions to address the needs of the diverse population. Future research should continue to explore these associations, focusing on the production of evidence and the implementation of policies to promote cardiovascular health, prevent the occurrence of CAD through longitudinal studies design, and incorporate objective measurements.

This study had significant aspects for healthcare practitioners, public health officials, and lawmakers. For instance, the rise in risk factor patterns such as physical inactivity, smoking, and obesity emphasizes the necessity of health initiatives that promote healthy behaviors through education, community participation, access to resources such as exercise programs, specific-risk prevention strategies designed for the need of the regions, and collaborative practices. Healthcare professionals must prioritize early detection, monitoring, and management to avoid the development of CAD, while policymakers must address socioeconomic gaps in healthcare access to remove barriers that prevent persons from accessing proper medical care services.

This review provides valuable insight into the current situation in Saudi Arabia. However, limitations have been identified, such as variability in study designs and methodologies throughout the included research. This makes it difficult to draw definitive conclusions. Furthermore, the majority of research relies on self-reported data, which may entail bias in reflecting behaviors such as smoking or eating. The cross-sectional character of several studies limits their capacity to provide casual relationships between risk factors and CAD outcomes. Including only English language studies could provide bias in the identification of studies. Future studies should overcome this limitation by including studies in other languages and more research designs.

Despite these limitations, this study synthesizes recent studies to allow for a better knowledge of the current situation in Saudi Arabia. Furthermore, this study provides insights into modifiable and non-modifiable risk variables, offering a better understanding of the complexity of CVD and the need for multifaceted prevention and management measures. Furthermore, the inclusion of a diverse set of research from various regions contributes to capturing the complexity of risk variables and their implications for different populations. This range of information helps provide a more comprehensive picture of Saudi Arabia's cardiovascular health environment and guides focused treatments.

## Conclusions

This extensive investigation identified important risk factors for CAD, emphasizing the significance of targeted public health interventions and integrated healthcare methods such as educational lectures and coordinated care models. Modifiable risk factors such as smoking, obesity, and chronic illnesses necessitate comprehensive programs that promote healthy lifestyles and habits.

The findings provide important information and insights into the current condition of CAD in Saudi Arabia, highlighting the need for considerable initiatives to overcome the limitations of existing research, such as systematic review and meta-analysis, by conducting more studies using a variety of components in addition to typical techniques. A better understanding of the interplay of CAD risk factors can help healthcare practitioners and policymakers to develop measures that improve health outcomes while reducing disease burden through public health policy interventions.

## Appendices

Search Strategy

Google Scholar

("acute coronary disease" OR "acute coronary syndrome" OR "coronary artery disease") AND ("risk factors" OR "determinants") AND ("Saudi Arabia" OR "KSA")

PubMed

("acute coronary disease"[All Fields] OR "acute coronary syndrome"[MeSH Terms] OR "coronary artery disease"[MeSH Terms]) AND (("risk factors"[MeSH Terms] OR "determinants"[All Fields]) AND ("Saudi Arabia"[MeSH Terms] OR "KSA"[All Fields]))

Web of Science

TS=("acute coronary disease" OR "acute coronary syndrome" OR "coronary artery disease") AND TS=("risk factors" OR "determinants") AND TS=("Saudi Arabia" OR "KSA")
